# Bilateral cerebral peduncle infarct in a paediatric intensive care unit patient

**DOI:** 10.4102/sajr.v30i1.3433

**Published:** 2026-04-14

**Authors:** Fatimah Sheik, Rabia Abid

**Affiliations:** 1 Department of Radiology, Faculty of Health, Chris Hani Baragwanath Hospital, Johannesburg, South Africa

**Keywords:** isolated bilateral cerebral peduncle infarction, rare stroke, paediatric stroke, Mickey Mouse sign, vertebrobasilar insufficiency, perforator arteries of the cerebral peduncles, paediatric stroke management

## Abstract

Bilateral isolated cerebral peduncle infarction is a rare stroke, previously reported only in adults. This report describes a paediatric burns patient with preceding cardiac arrest who developed subtle neurology. Imaging revealed bilateral isolated peduncle infarcts with hypoplasia of the left posterior communicating and vertebral arteries. Despite supportive care, the patient deteriorated and demised. This case highlights the vulnerability of peduncular perforators to haemodynamic instability and emphasises the need for early imaging in the detection of paediatric stroke.

**Contribution:**

Isolated bilateral cerebral peduncle infarction is a rare form of stroke, particularly in the paediatric population, and is associated with a poor prognosis. Imaging plays an important role in the early diagnosis of this type of stroke.

## Introduction

Isolated bilateral crus cerebri infarction is a rare form of ischaemic stroke.^1^ The cerebral peduncles, located in the anterior midbrain, contain major white matter tracts, including the corticospinal, corticobulbar and corticopontine pathways.^2^ Most peduncular infarcts occur within recognised syndromes, such as Weber syndrome, or with infarction of adjacent structures, including the thalamus, pons or cerebellum.^3,4,5^ To the authors’ knowledge, isolated bilateral cerebral peduncle infarction has previously only been reported in adults from Asia and Europe.^1,3,6^ This case represents the first paediatric report from Africa and highlights the critical role of MRI in early diagnosis.

## Patient presentation

A previously healthy 19-month-old female was admitted with 20% superficial, partial-thickness burns to the head, back and limbs, requiring intravenous fluids, wound care, analgesia and rehabilitation. Her hospital course was complicated by poor wound healing and sepsis, with initial blood cultures growing coagulase-negative *Staphylococcus*. Despite multiple sloughectomies, the wounds remained unhealed, and she developed further nosocomial infections, including *Candida albicans* (fluconazole-sensitive), multidrug-resistant *Acinetobacter baumannii, Staphylococcus aureus* (vancomycin-sensitive) and *Pseudomonas aeruginosa* (ciprofloxacin-sensitive). The patient deteriorated despite targeted therapy, developing septic shock with multiorgan failure and cardiac arrest, from which she was successfully resuscitated. She required intubation and ventilation with escalation of supportive care, including fluid management, nutritional support, albumin supplementation, antibiotics, steroids, analgesia, seizure and ulcer prophylaxis and daily organ profile monitoring.

On day 29 post-cardiac arrest, the patient developed new neurological deficits, with reduced responsiveness (GCS 6T/10- E2M4VT), impaired visual tracking and bilateral pupillary hyporeactivity. There was global weakness, greater in the upper limbs than in the lower limbs. Reflexes were brisk globally (3/4) with upgoing plantars. Tactile sensation and proprioception were intact, and no seizures were observed.

**FIGURE 1:**
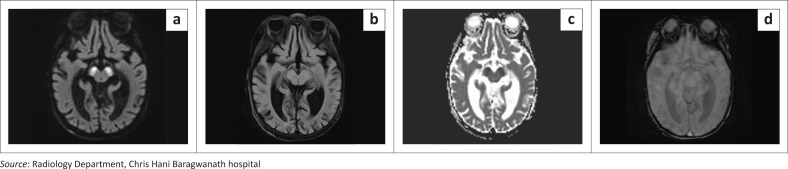
(a) Diffusion-weighted axial MRI of the brain at the level of the cerebral peduncles, showing bilateral hyperintense signals in the cerebral peduncles, the ‘Mickey Mouse sign’. (b) T2 FLAIR axial MRI of the brain showing subtle hyperintense signals in both cerebral peduncles, indicating an acute to subacute infarct more than 4.5 h later. (c) Apparent diffusion coefficient axial MRI of the brain, showing corresponding hypointensity in keeping with restricted diffusion in the region of the cerebral peduncles. (d) SWI axial MRI shows no acute haemorrhagic component in the bilateral cerebral peduncle infarcts.

**FIGURE 2:**
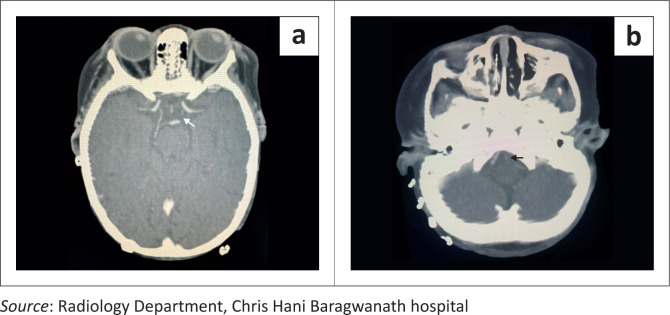
(a) Left posterior communicating artery hypoplasia. (b) Left vertebral artery hypoplasia.

MRI performed 4 days later demonstrated bilateral, symmetrical, isolated, hyperintense lesions in the cerebral peduncles on DWI and T2-weighted FLAIR sequences, with corresponding ADC restriction, consistent with acute ischaemia, and absence of a haemorrhagic component on SWI (Figure 1). No additional infarcts were identified. CT angiography revealed hypoplastic left vertebral and posterior communicating arteries (Figure 2), without evidence of acute arterial occlusion or vascular malformation. No acute antithrombotic therapy was initiated, and supportive care was continued. The patient underwent split-thickness skin grafting the following day without clinical deterioration. On day 18 post-surgery, the infant suffered a sudden cardiac arrest, was unsuccessfully resuscitated and pronounced deceased.

## Discussion

**FIGURE 3:**
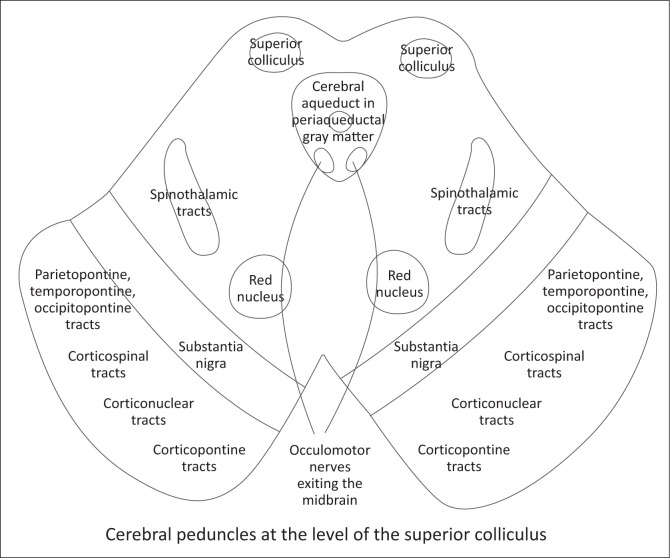
Illustration of the cerebral peduncles with compartmentalisation of the white matter tracts.

The cerebral peduncles have a compartmentalised anatomy (Figure 3). The paramedian region lies most medially, where the oculomotor nerve exits the brainstem.^6,7^ Fronto-pontine fibres occupy the medial portion, whilst the corticospinal tracts comprise the central three-fifths of the peduncle and are arranged somatotopically, with arm fibres medially, followed by face and leg fibres laterally.^5,6^ Parieto-occipito-pontine fibres occupy the lateral portions. The corticobulbar tracts lie dorsomedial to the corticospinal tracts and project to the cranial nerve nuclei.^5,6^ Spinothalamic fibres are located dorsolaterally within the medial lemniscus and mediate pain, temperature and crude touch sensation.^6^

The MRI findings in this case align with prior reports of isolated acute bilateral cerebral peduncle infarctions. The T2, FLAIR and DWI sequences show hyperintense signals in the peduncles, dubbed the ‘Mickey Mouse sign’,^1,6^ whilst ADC sequences demonstrate corresponding hypointensity.^1,3,4,5^ Kwon et al.^1^ used diffusion tensor imaging and fibre tractography to visualise white matter tracts, but even in its absence, the infarct was accurately localised based on neurological examination and MRI findings.

The complex neuroanatomy of the peduncles signifies that even a small area of infarct can lead to a poor prognosis.^1^ In older published case reports, patients presented with the locked-in syndrome or vegetative state,^1,3^ often culminating in death. In more recent cases, more varied presentations were encountered, some of which presented with milder, although not without, debilitating symptoms. These include altered consciousness,^3,4^ quadriplegia,^4^ pseudobulbar palsy,^1,3,6^ ataxia^3,5,6^ and a positive Babinski’s reflex.^3,5^ Pseudobulbar palsy signs include cognitive impairment,^6^ dysarthria, dysphagia, drooling, facial weakness or paresis, dysphonia and emotional changes such as uncontrollable laughter or crying.^1,5^ Chen et al.^3^ found in their retrospective analysis of 11 patients that 81.8% had consciousness disturbance, 63.6% had quadriplegia, and 27.3% had pseudobulbar palsy.

In paediatric patients, neurological signs may be subtle, and stroke is an uncommon cause, accounting for fewer than one-third of cases.^8^ Imaging therefore plays a critical role in the early diagnosis of ischaemic infarction. The patient in this report presented with long tract signs, including global weakness with predominant upper-limb involvement, brisk reflexes and bilateral upgoing plantar responses, consistent with bilateral medial corticospinal tract involvement within the mid-crus cerebri. Ocular findings suggest involvement of the most medial aspects of the cerebral peduncles. The oculomotor nerve courses along the medial border of the peduncles before exiting the midbrain via the superior orbital fissure.^7^ The patient demonstrated impairment of both voluntary and parasympathetic oculomotor fibres, evidenced by absent tracking and reduced pupillary responses, likely reflecting parasympathetic fibre damage.^7^ Preservation of eye opening to pain may reflect compensatory activation of other muscles involved in eyelid elevation, such as the frontalis muscle.^9^

The cerebral peduncles are supplied predominantly by the posterior circulation. The primary blood supply arises from paramedian branches of the P1 and P2 segments and circumferential branches of the posterior cerebral artery, supplying the medial and ventral aspects, with additional contribution from paramedian branches of the basilar artery.^10,11^ Other contributors include the superior cerebellar artery, branches of the posterior communicating arteries, and occasionally the anterior choroidal artery from the anterior circulation.^11^ Case reports using MRA and CTA most commonly demonstrate bilateral or midline proximal large-vessel pathology of the posterior circulation, including vertebrobasilar occlusion or stenosis and/or bilateral posterior cerebral artery occlusion.^1,3,4,6^ the underlying cause is typically confirmed to be large-vessel atherothrombotic disease associated with hypertension, diabetes mellitus and smoking,^1,2,3,4,5,6^ although cardioembolic and artery-to-artery embolic causes involving the basilar or posterior cerebral arteries have also been reported. In contrast, paediatric arterial ischaemic stroke has distinct aetiologies, including arteriopathies, congenital heart disease, chronic systemic infections, particularly varicella, and prothrombotic states, with sepsis recognised as a contributing factor.^12^

Guideline-recommended investigations for these causes are extensive, underscoring the need for evidence-based paediatric stroke protocols specific to under-resourced settings. For emergency imaging, MRI (standard sequences, DWI and SWI) is the gold standard, supplemented by MRA of the brain and cervical vessels or CTA from the aortic arch to the vertex. In this case, brain CTA revealed no haemodynamically significant stenosis or occlusion of the large arteries of the posterior or anterior circulation. CTA would not have provided sufficient resolution to detect the pathology of the small perforator arteries supplying the cerebral peduncles. CTA demonstrated hypoplastic left posterior communicating and vertebral arteries, which are normal variants. Evidence on whether hypoplastic cerebral arteries increase stroke risk is limited and inconsistent,^13,14^ which precludes definitive causal conclusions.^15,16,17^ We presently support the hypothesis proposed in case reports^1,5^ that the terminal branches supplying the cerebral peduncles are particularly vulnerable to haemodynamic instability. In the presented case, prior cardiac arrest and prolonged sepsis rendered the perforator arteries of the cerebral peduncles vulnerable to ischaemic insult.

There is no published evidence guiding the specific management of cerebral peduncle infarctions. Reported adult cases did not receive acute thrombolysis in line with existing stroke guidelines,^18^ but were managed with antithrombotic or antiplatelet therapy,^3,5^ cerebral oedema control, risk factor modification, seizure management and early rehabilitation for pseudobulbar palsy and limb weakness.^3,5^ Outcomes were generally poor, with high early mortality or severe disability, including quadriparesis, gait impairment, dysarthria and ophthalmoplegia requiring prolonged rehabilitation.^3,4^ Clear guidelines for acute paediatric stroke management are limited.^8,12^ Current recommendations discourage intravenous or mechanical reperfusion therapies in children under 2 years, whilst aspirin is considered safe despite limited efficacy data. Management is largely supportive and mirrors adult protocols, emphasising airway and ventilation optimisation (SpO_2_ > 94%, PaCO_2_ 35–45 mmHg), haemodynamic stability avoiding hypotension, euvolaemia, normoglycaemia, normothermia and treatment of seizures when present; routine prophylactic antiseizure therapy is not recommended. Early specialist involvement is advised in selected cases, including posterior circulation infarction. Long-term care requires multidisciplinary rehabilitation.^8,12^ Despite supportive management, the presented patient did not survive the neurological insult.

## Conclusion

Isolated bilateral cerebral peduncle infarction is an exceptionally rare and devastating form of stroke. This case represents, to our knowledge, the first reported paediatric case from Africa and underscores the diagnostic value of MRI, including DWI, in detecting early posterior circulation infarction when clinical findings are subtle or non-specific. The vulnerability of the peduncular perforator arteries to haemodynamic compromise, particularly in the context of sepsis and cardiac arrest, may contribute to infarction in the absence of large-vessel occlusion. Greater awareness of this entity and its imaging features is essential, especially in resource-limited settings, to support early diagnosis, appropriate management and the development of paediatric stroke.
